# Rapid Molecular Diagnostics for Bloodstream Infection in Patients with Chronic Kidney Disease

**DOI:** 10.3390/diagnostics16081156

**Published:** 2026-04-14

**Authors:** Ayman Elbehiry, Eman Marzouk, Adil Abalkhail, Sulaiman Anagreyyah, Abdulrhman Almalki, Naif Alazwari, Hatim Ramza, Abdulilah Alsolami, Ayman Alghamdi

**Affiliations:** 1 Department of Public Health, College of Applied Medical Sciences, Qassim University, P.O. Box 6666, Buraydah 51452, Saudi Arabia; e.marzouk@qu.edu.sa (E.M.);; 2 Family Medicine Department, King Fahad Armed Hospital, Jeddah 23311, Saudi Arabia; 3 Cardiology Department, King Fahad Armed Forces Hospital, Jeddah 23311, Saudi Arabia; 4 Nephrology Department, King Fahad Armed Forces Hospital, Jeddah 23311, Saudi Arabia; 5 Radiology Department, King Fahad Armed Forces Hospital, Jeddah 23311, Saudi Arabia

**Keywords:** chronic kidney disease, hemodialysis, bloodstream infection, rapid molecular diagnostics, antibiotic resistance, genotype-phenotype concordance, antimicrobial stewardship

## Abstract

Bloodstream infection (BSI) is a major cause of morbidity and mortality in patients with chronic kidney disease (CKD), particularly those receiving hemodialysis. Delayed identification of pathogens and their resistance profiles can lead to inappropriate therapy and adverse outcomes. This review evaluates rapid molecular diagnostic approaches for detecting pathogens and resistance markers in BSI, with emphasis on their application in CKD. These technologies provide faster microbiological information by enabling direct or accelerated detection of pathogens and selected resistance determinants. Clinical studies indicate that their use supports prompt adjustment of antimicrobial therapy, especially when combined with antimicrobial stewardship and applied after blood culture positivity. In CKD, identification of the causative organism facilitates treatment selection aligned with renal function and helps reduce unnecessary exposure to nephrotoxic agents. However, diagnostic accuracy differs among platforms, and detection of resistance genes does not consistently reflect phenotypic susceptibility. Furthermore, most evidence is derived from mixed hospital populations rather than CKD-specific cohorts. These factors require careful interpretation within the clinical context. Rapid molecular diagnostics can enhance antimicrobial decision-making in BSI, but their effectiveness depends on integration with conventional microbiology and structured care pathways. Further research in CKD populations is required to clarify their impact on clinical outcomes and to support implementation in nephrology practice.

## 1. Introduction

Bloodstream infection (BSI) is defined as the presence of viable pathogenic microorganisms in the normally sterile bloodstream, confirmed by blood culture (BC) in a symptomatic patient [1]. It is a microbiological diagnosis and differs from sepsis, which is defined as life-threatening organ dysfunction caused by a dysregulated host response to infection [2]. Although distinct, BSI may precede sepsis when microbial invasion triggers systemic injury. Bacteria account for most cases, while fungal pathogens such as *Candida* species contribute substantially among immunocompromised individuals and patients with intravascular devices [3,4].

Chronic kidney disease (CKD) is defined by structural or functional kidney abnormalities lasting more than three months and affects a substantial proportion of adults worldwide [5]. It is associated with increased infection-related morbidity and mortality. In a large population-based cohort, individuals with an estimated glomerular filtration rate below 30 mL/min/1.73 m^2^ had a hazard ratio of 3.54 for community-onset BSI and 4.10 for 30-day mortality compared with those with preserved kidney function [6]. These findings show that declining kidney function increases both susceptibility to BSI and the risk of death once infection develops.

The risk is even higher in patients receiving maintenance dialysis. In a nationwide Danish cohort, patients undergoing hemodialysis (HD) had an approximately 26-fold higher incidence of BSI than matched population controls [7]. Catheter-related BSIs remain common and are associated with prolonged hospitalization and serious complications [8]. Case fatality rates range from 19% to 25%, reflecting the severity of these infections [9]. Mortality attributed to sepsis is also markedly higher in patients with end-stage renal disease receiving dialysis than in the general population [10].

This increased risk reflects both biological vulnerability and treatment-related exposure. CKD impairs innate and adaptive immune responses, including neutrophil and lymphocyte function [11,12]. Repeated vascular access for HD, particularly through central venous catheters, provides a direct route for microbial entry into the bloodstream [13]. These factors create conditions in which BSI is frequent and clinically severe.

Diagnosis still relies mainly on BC followed by phenotypic antimicrobial susceptibility testing (AST). BC remains the reference standard because it allows organism isolation and resistance assessment [1]. Definitive identification and susceptibility results usually require 24 to 72 h or longer, even with automated systems [14,15]. Delayed administration of effective antimicrobial therapy has been independently associated with increased mortality in BSI [16,17]. In CKD, limited renal reserve further increases vulnerability to both infection-related injury and drug toxicity, making delays in appropriate therapy particularly harmful [6,7].

High infection risk, increased mortality, and diagnostic delay create a major challenge in nephrology practice [6,18]. Rapid and accurate identification of pathogens and their antimicrobial resistance (AMR) determinants is essential to guide timely and kidney-safe therapy [14,15]. Molecular diagnostic technologies aim to shorten the interval between clinical suspicion and microbiologic confirmation and support earlier targeted antimicrobial treatment.

Although several reviews have evaluated rapid diagnostics in general sepsis populations, limited attention has been given to their use in the specific clinical context of CKD. Patients with CKD present distinct challenges related to immune dysfunction, dialysis-related exposure, altered pathogen profiles, and increased susceptibility to antimicrobial toxicity. These factors may influence both diagnostic performance and therapeutic decisions. A focused nephrology perspective is therefore needed to determine how molecular diagnostics can be applied safely and effectively in this high-risk population.

The aim of this review was to provide a nephrology-focused synthesis of rapid molecular diagnostics for bloodstream infection in patients with CKD. It integrates evidence on diagnostic performance, antimicrobial resistance detection, and clinical application within the context of renal dysfunction. It also emphasizes antimicrobial optimization and renal safety, while highlighting current limitations, variability in evidence, and key gaps specific to CKD populations.

## 2. Methodology

### 2.1. Study Design

This study was conducted as a structured narrative review to synthesize current evidence on rapid molecular diagnostics for BSI in patients with CKD. It integrates clinical studies, diagnostic evaluations, and translational research within a nephrology-focused context.

This review does not follow a formal systematic review or meta-analysis. Methodological rigor was maintained through a predefined search strategy, clear eligibility criteria, and structured study selection. Studies were selected based on clinical relevance rather than strict quantitative thresholds, consistent with a narrative approach.

### 2.2. Search Strategy

A literature search was conducted using PubMed, Scopus, and Web of Science. Publications from 2000 to 2026 were included to capture the evolution of molecular diagnostic technologies from early amplification methods to current sequencing approaches.

Search terms combined controlled vocabulary and free-text keywords related to BSI, bacteremia, sepsis, CKD, HD, molecular diagnostics, multiplex polymerase chain reaction (multiplex PCR), droplet digital PCR (ddPCR), metagenomic sequencing, and AMR. Search syntax was adapted for each database. Where applicable, MeSH and database-specific terms were used to improve sensitivity.

**Figure 1:**
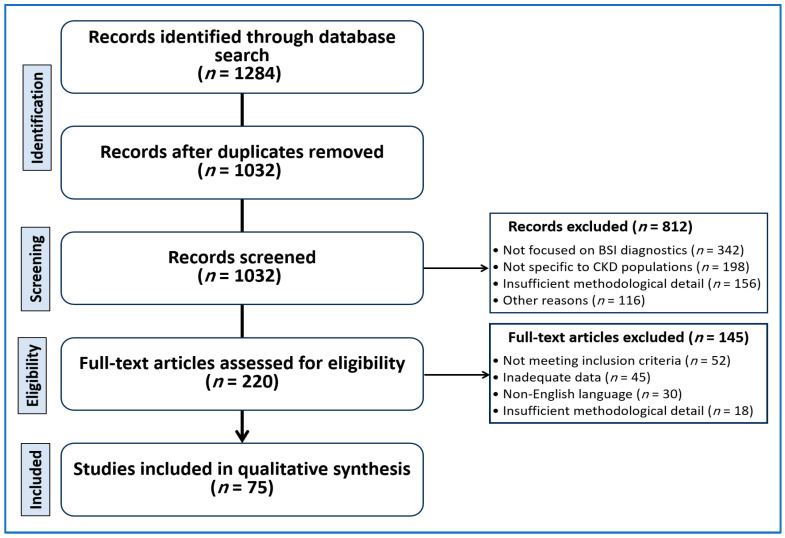
Study selection flow adapted from PRISMA principles. The diagram outlines the identification, screening, eligibility assessment, and inclusion of studies forming the qualitative synthesis.

Only English-language articles were included. Reference lists of relevant studies and reviews were screened to identify additional sources. The search identified 1284 records, of which 1032 remained after duplicate removal. The study selection process is summarized in Figure 1 using a PRISMA-adapted framework. The strategy aimed to capture both supportive and cautionary evidence related to diagnostic performance and clinical application.

### 2.3. Study Selection

Study selection was conducted in two stages: screening of titles and abstracts followed by full-text review. Two reviewers independently assessed eligibility, and disagreements were resolved by consensus.

Eligible studies included randomized trials, prospective and retrospective studies, diagnostic accuracy studies, and systematic reviews addressing molecular diagnostics in BSI or AMR. Studies reporting clinical outcomes, time to appropriate therapy, or antimicrobial stewardship were included.

Exclusion criteria included case reports, narrative commentaries without primary data, conference abstracts lacking methodological detail, and studies not directly relevant to BSI diagnostics or AMR.

Exclusion criteria included case reports, narrative commentaries without primary data, conference abstracts lacking methodological detail, and studies not directly relevant to BSI diagnostics or AMR. Selection was based on relevance to the scope of the review rather than strict quantitative thresholds.

Studies involving CKD or HD populations were prioritized. When CKD-specific data were limited, evidence from broader hospitalized or sepsis populations was included and interpreted cautiously within a nephrology context.

After full-text review, 75 primary studies were included in the qualitative synthesis. Additional reviews and guidelines supported interpretation. The study selection process is summarized in Figure 1.

### 2.4. Data Extraction and Evidence Synthesis

Data extraction focused on diagnostic performance, resistance detection, turnaround time (TAT), and clinical outcomes. Differences between molecular platforms and their clinical applicability were also examined.

Evidence was interpreted according to the study design. Greater weight was given to randomized trials, prospective studies, and systematic reviews, while observational and analytical studies were interpreted with caution.

The synthesis followed a structured thematic framework covering diagnostic performance, resistance detection, clinical impact, and implementation considerations.

Due to heterogeneity in study design and outcome reporting, quantitative analysis was not performed. Findings were synthesized qualitatively, with attention to differences in methodology and consistency of results.

### 2.5. Consideration of Bias and Limitations

This review is subject to limitations inherent to a structured narrative synthesis. A formal risk-of-bias assessment using standardized tools was not performed, and this may limit the ability to systematically evaluate the internal validity of included studies. Study quality was assessed qualitatively during interpretation.

Potential sources of bias include selection bias in observational studies, limited generalizability of single-center data, and possible overestimation of diagnostic performance in controlled settings. In addition, the absence of predefined quantitative inclusion thresholds may introduce subjective selection bias and favor studies with positive findings. Greater weight was given to higher-quality evidence, while lower-quality studies were interpreted cautiously.

Most available data were derived from heterogeneous hospital populations rather than CKD-specific cohorts. Where non-CKD data were included, findings were interpreted in light of differences in clinical context and antimicrobial exposure. This limits the direct applicability of the findings to nephrology practice, where patient characteristics and infection patterns may differ.

Variation in diagnostic performance across platforms and study settings was considered to avoid overgeneralization. However, substantial heterogeneity in study design, patient populations, and outcome definitions limits direct comparison between studies and prevents quantitative synthesis. Therefore, the conclusions of this review should be interpreted as evidence-informed rather than definitive.

## 3. Pathophysiological Basis of Infection Susceptibility in CKD

### 3.1. Innate Immune Dysfunction

CKD disrupts innate immune defense through accumulation of uremic solutes caused by reduced renal clearance. Protein-bound toxins such as indoxyl sulfate and p-cresyl sulfate originate from intestinal microbial metabolism and accumulate as kidney function declines. These compounds promote oxidative stress and persistent inflammatory signaling, which interfere with normal immune cell activity [19].

Neutrophil function is markedly altered in advanced CKD. Key processes, including chemotaxis, phagocytosis, and intracellular killing, are reduced. Changes in oxidative burst activity have been linked to altered NADPH oxidase signaling [20]. These defects weaken early microbial control and increase the likelihood that transient bacteremia progresses to sustained infection.

Monocyte and macrophage responses are also modified. Expansion of pro-inflammatory CD14++CD16+ monocyte subsets leads to increased baseline cytokine production, but coordinated antimicrobial activity is less effective [21]. This imbalance promotes persistent inflammation without adequate pathogen elimination.

Complement pathways are similarly affected. Altered activation and consumption reduce opsonization efficiency and impair pathogen recognition [22]. As a result, containment of circulating microorganisms becomes less effective.

These changes delay early microbial clearance from the bloodstream. Even low-level bacteremia may persist, increasing the probability of sustained infection and systemic inflammatory injury in patients with CKD and those receiving HD [12,18,19].

### 3.2. Adaptive Immune Dysfunction

Adaptive immunity in CKD exhibits features of premature immunosenescence. Patients with advanced disease, particularly those undergoing dialysis, show reduced naïve T-cell production and decreased T-cell receptor diversity [23]. At the same time, expansion of terminally differentiated T cells expressing inhibitory receptors such as programmed cell death protein 1 has been observed [24]. These changes limit effective clonal expansion and weaken pathogen-specific responses.

Functional impairment extends beyond cell numbers. Disrupted cytokine signaling and chronic inflammatory activation interfere with coordinated immune responses [21]. This shift favors persistent immune activation without effective control of infection.

B-cell function is also affected. Reduced memory B-cell formation and impaired class-switch recombination lead to weaker antibody responses following infection or vaccination [19,23]. Both the magnitude and functional quality of humoral immunity are diminished.

Accordingly, pathogen clearance is less efficient and long-term immune protection is limited. Patients with CKD remain susceptible to recurrent bacteremia and device-related infections, particularly in dialysis settings with repeated microbial exposure [25,26].

### 3.3. Treatment-Related and Extrinsic Factors

In addition to intrinsic immune changes, treatment-related exposures further increase infection risk. Long-term vascular access for HD, especially central venous catheters, provides direct entry for microorganisms into the bloodstream [27]. Biofilm formation on catheter surfaces allows microbial persistence and reduces susceptibility to host defenses and antimicrobial therapy [27].

Frequent healthcare exposure promotes colonization with resistant organisms. Patients undergoing dialysis have repeated contact with healthcare environments, facilitating acquisition of multidrug-resistant pathogens [28]. Recurrent antimicrobial use creates selective pressure that favors resistant strains. Dialysis procedures may also contribute to ongoing inflammatory activation through complement and leukocyte stimulation [26].

Common comorbid conditions further compromise host defense. Diabetes, malnutrition, metabolic acidosis, vitamin D deficiency, and protein-energy wasting weaken epithelial barriers and immune competence [21]. These factors lower resistance to invasive infection.

The combined effects of immune alteration and repeated procedural exposure increase both the frequency and severity of BSI in CKD. As kidney function declines and dialysis dependence develops, susceptibility to infection becomes more pronounced [18,29,30].

**Figure 2:**
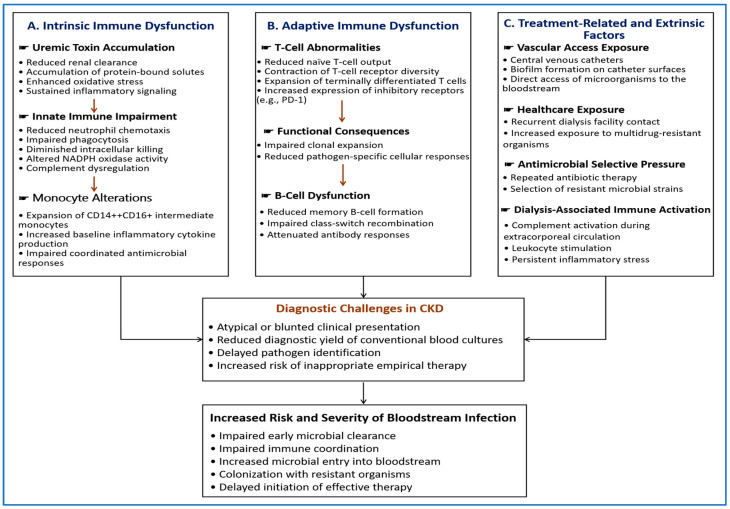
Mechanisms underlying increased susceptibility to BSI in patients with CKD and their diagnostic implications. Intrinsic and adaptive immune dysfunction, together with treatment-related and extrinsic factors, impair host defense and promote microbial entry. These alterations contribute to atypical clinical presentation, reduced diagnostic yield of conventional BCs, and delayed pathogen identification. Consequently, timely and appropriate antimicrobial therapy may be compromised, leading to increased risk and severity of BSI in this population.

These mechanisms and their diagnostic implications are summarized in Figure 2. They contribute to delayed pathogen detection and reduced sensitivity of conventional diagnostics, supporting the role of rapid molecular approaches in this setting.

Clinical studies support these observations. Declining kidney function has been associated with a higher risk of BSI, and bacteremia remains a major clinical burden in HD populations [6,18]. Prior antimicrobial exposure reduces BC positivity by suppressing viable organisms [31,32]. Molecular diagnostic methods have demonstrated earlier pathogen detection compared with conventional culture-based techniques [33,34,35].

## 4. Limitations of Conventional Diagnostic Approaches in CKD-Associated BSI

### 4.1. Time Delays and Sensitivity Limitations of BC

BC enables recovery of viable microorganisms for identification and phenotypic AST [1,36]. Detection depends on microbial replication within culture bottles, and time to positivity varies with organism load and growth characteristics, often requiring 24 to 72 h or longer [3,36,37].

Sensitivity is influenced by both clinical and technical factors, particularly prior antimicrobial exposure. Antibiotic administration before sampling reduces culture yield because detection depends on viable organisms [31,32]. Consequently, cultures obtained after treatment initiation may remain negative despite ongoing infection.

Organism-specific characteristics further affect detection. *Candida* species often show lower sensitivity and longer time to positivity than common bacterial pathogens [38]. Reported sensitivity for candidemia ranges from approximately 50% to 75%, and invasive candidiasis may occur with negative cultures when fungal burden is low or infection is deep-seated [39].

Sampling factors also influence yield. Adequate blood volume and multiple culture sets improve detection, whereas single or low-volume samples reduce sensitivity [40]. Intermittent or low-level bacteremia may therefore go undetected. In dialysis settings, frequent antimicrobial exposure and healthcare contact may further reduce culture yield and contribute to diagnostic uncertainty [18,41].

### 4.2. Resistance Characterization and Therapeutic Implications

Following culture positivity, additional processing is required for organism identification and susceptibility testing. Subculture and incubation typically add 24 to 48 h before resistance profiles become available [42]. During this period, treatment decisions rely on empirical therapy.

In BSI, delayed administration of effective therapy has been associated with increased mortality and longer hospital stays [43,44]. Although rapid diagnostic approaches can shorten time to appropriate therapy [14,15], conventional methods provide resistance data only after organism growth and additional processing.

Empirical broad-spectrum therapy is commonly initiated in suspected BSI [45]. In CKD, this approach may increase exposure to nephrotoxic agents such as aminoglycosides or vancomycin, which are associated with drug-induced kidney injury [46,47]. Unrecognized resistance can lead to ineffective initial therapy, while prolonged empirical treatment may promote antimicrobial resistance [17,48]. Delayed optimization therefore affects both microbiologic control and treatment safety.

### 4.3. Diagnostic Challenges in Dialysis and Device-Related Infection

In patients receiving HD, vascular access devices introduce additional complexity. Central venous catheters provide a direct route for microbial entry into the bloodstream [27]. Microorganisms can migrate along the catheter tract or enter through hub contamination during routine handling [49,50].

Biofilm formation on catheter surfaces allows organisms to persist and intermittently enter the circulation [51]. This pattern can result in transient or low-level bacteremia, reducing the sensitivity of culture-based detection.

Interpretation of positive cultures may also be difficult. Coagulase-negative staphylococci (CNS) are common contaminants but are also recognized causes of catheter-related infection [36,50]. Distinguishing contamination from true infection requires multiple cultures and clinical correlation [1].

Frequent healthcare exposure and repeated antimicrobial use increase colonization with multidrug-resistant organisms, including MRSA and ESBL-producing Enterobacterales [52]. Confirmation of susceptibility using conventional methods requires additional time after culture positivity, which may postpone targeted therapy in high-risk patients [53,54,55].

Conventional culture remains essential but requires time for organism growth and susceptibility testing [1,36]. In CKD and HD populations, where infection-related mortality is elevated, delays in pathogen identification and resistance confirmation can have significant clinical consequences [56]. Despite these limitations, culture-based methods remain essential for organism recovery and susceptibility testing. Their constraints are summarized in Table 1.

## 5. Molecular Diagnostic Approaches for Rapid Pathogen and Resistance Detection

Evidence supporting molecular diagnostic platforms is derived from studies with varying methodological quality. Prospective clinical investigations and randomized trials provide the most reliable data on clinical impact, particularly regarding time to appropriate antimicrobial therapy [14,15]. In contrast, much of the analytical performance data for emerging technologies, including ddPCR and sequencing-based methods, originates from controlled experimental settings or mixed patient populations [57,58,59].

Rapid identification of pathogens and their AMR determinants plays a central role in BSI management. Conventional BC requires organism growth before identification and susceptibility results are available, whereas molecular and accelerated methods shorten this process by detecting microbial genetic material directly or by expediting identification after culture positivity [36,60]. Integration of these approaches into clinical workflows has been associated with earlier therapeutic adjustment when combined with antimicrobial stewardship [14,15]. The main platforms are summarized in Table 2.

### 5.1. Direct-from-Blood Molecular Assays

Direct-from-blood assays use nucleic acid amplification techniques, most commonly multiplex real-time PCR, to detect pathogen DNA or RNA without prior culture. Results are typically available within hours. Clinical sensitivity depends on pathogen burden, sample volume, extraction efficiency, and background host DNA [3,33,34,61].

Multiplex systems target predefined panels of organisms. They perform well for common pathogens but cannot detect organisms outside the panel, limiting diagnostic scope [34,61].

ddPCR allows absolute quantification of microbial DNA and shows high analytical sensitivity in controlled studies [57]. However, clinical validation remains limited. Resistance gene detection is possible with targeted primers, but agreement with phenotypic susceptibility is not fully established [58]. Pre-analytical variables, including sample handling and extraction, also influence results. Current evidence supports its role as an adjunct rather than a replacement for culture-based methods.

Reported sensitivity varies across studies due to differences in design, patient selection, and laboratory processes [33,34,35,61]. Many evaluations involve selected or critically ill populations, which may limit generalizability to routine nephrology practice.

### 5.2. Rapid Identification and Susceptibility Testing from Positive BCs

Once BC becomes positive, accelerated identification methods reduce laboratory turnaround time. MALDI-TOF mass spectrometry provides species-level identification directly from culture broth and supports earlier modification of antimicrobial therapy when integrated with clinical care [62,63].

Multiplex PCR panels applied to positive BCs identify common pathogens and selected resistance genes within approximately one hour [14,64]. These platforms enable earlier adjustment of therapy and reduce unnecessary broad-spectrum antibiotic use when incorporated into clinical workflows [14].

Rapid susceptibility testing performed directly from positive cultures further shortens the time to actionable results. The EUCAST rapid AST method provides categorical susceptibility data within 4 to 8 h and shows acceptable agreement with standard testing [65]. Combining rapid identification with early susceptibility assessment facilitates timely therapeutic modification [66].

Clinical benefit is most consistent when results are interpreted within structured stewardship programs, which support appropriate antimicrobial selection [15,62].

### 5.3. Next-Generation and Emerging Molecular Platforms

Next-generation sequencing (NGS) enables culture-independent detection of microbial DNA or RNA without predefined targets. Shotgun metagenomic approaches allow identification of bacterial, fungal, viral, and parasitic organisms within a single analysis [59,67].

Plasma microbial cell-free DNA sequencing has demonstrated pathogen detection in patients with suspected infection, including cases with negative BCs, particularly after prior antimicrobial exposure [68]. Diagnostic yield varies depending on sequencing depth, analytical methods, and patient characteristics [69].

Sequencing-based techniques can identify resistance genes; however, genetic detection does not consistently correspond to phenotypic susceptibility. Interpretation requires caution due to variability in gene expression and incomplete coverage of resistance mechanisms [70]. Additional challenges include host DNA interference, cost, bioinformatic complexity, and longer processing time compared with targeted assays [59].

Most available studies are observational, and direct comparisons with conventional diagnostics remain limited. Current use is therefore largely adjunctive or reserved for complex or unresolved cases.

### 5.4. Clinical Integration and Resistance Interpretation in CKD

The clinical value of molecular diagnostics depends on their incorporation into coordinated care pathways. Meta-analyses show that combining rapid diagnostics with antimicrobial stewardship leads to earlier initiation of effective therapy and improved antimicrobial optimization [15].

In CKD, antimicrobial therapy must be adjusted to account for altered drug clearance and risk of toxicity. Earlier pathogen identification supports narrowing of therapy and reduction of unnecessary exposure to nephrotoxic agents [71].

Detection of resistance genes does not always reflect phenotypic susceptibility. Differences in gene expression, regulatory mechanisms, and panel coverage contribute to discordance between molecular findings and conventional testing [72,73]. Misinterpretation may lead to unnecessary escalation if molecular results are considered in isolation.

False-positive findings, including detection of non-viable DNA or contamination, have been reported, particularly with sequencing-based methods. Clinical interpretation therefore requires integration with microbiological results and patient context.

Most studies involve heterogeneous hospital populations, and applicability to CKD-specific settings remains uncertain.

**Table 2:** Molecular diagnostic platforms for BSI: evidence-based characteristics.

Platform	Sample Type	Detection Scope	Resistance Detection Capability	Time to Result	Key Evidence Statement	Clinical Role in BSI Management	References
Multiplex PCR (Direct-from-Blood)	Whole blood	Predefined panel of common bacterial and fungal pathogens	No phenotypic susceptibility testing; limited resistance gene targets based on panel design	Several hours after sample collection	Feasible for direct detection; sensitivity varies with pathogen burden	Adjunct diagnostic tool in high-risk or culture-negative patients; not a replacement for BC	[33,34]
Broad-Range PCR (Direct-from-Blood)	Whole blood	Bacterial DNA detection using conserved gene targets	No routine resistance profiling	Variable; dependent on extraction method and laboratory workflow	Detects pathogens in selected sepsis cases; affected by extraction efficiency and contamination	Useful in selected culture-negative infections; interpretation requires clinical context	[61]
ddPCR	Whole blood (primarily analytical studies)	Target-specific pathogen detection with absolute quantification	Targeted resistance gene detection in analytical studies; clinical validation limited	Variable; dependent on workflow	High analytical sensitivity; clinical validation in BSI remains limited	Adjunct method; limited data on impact on clinical decision-making	[57,58]
MALDI-TOF MS (from Positive BC)	Positive BC broth	Species-level organism identification	Does not provide phenotypic susceptibility; requires separate AST	Same-day identification after culture positivity	Shortens time to identification and therapy when integrated with stewardship	Standard method for rapid identification after BC positivity; supports early therapy adjustment	[62,63]
Multiplex PCR (from Positive BC)	Positive BC broth	Identification of common bacterial and fungal species; selected resistance genes	Detection of predefined resistance markers; no full phenotypic susceptibility	Approximately 1 h after culture positivity	Reduces time to optimal therapy and unnecessary broad-spectrum antibiotic use	Supports early targeted therapy after organism identification	[14,64]
Rapid AST (EUCAST RAST)	Positive BC isolate	Phenotypic susceptibility testing using shortened incubation	Provides early categorical susceptibility results; limited to validated organism-antibiotic combinations	4–8 h after culture positivity	Moderate agreement with standard AST; provides earlier actionable results	Provides early phenotypic susceptibility results to guide antimicrobial therapy	[65]
Metagenomic NGS (mNGS)	Plasma or whole blood	Culture-independent detection of bacterial, fungal, viral, and parasitic DNA	Detection of resistance genes; phenotypic correlation incomplete	Typically, longer than targeted PCR; workflow dependent	Detects pathogens in culture-negative cases; performance depends on sequencing depth and analysis	Adjunct tool in complex or culture-negative cases; requires careful interpretation	[59,68]

## 6. Clinical Impact and Translational Integration of Rapid Molecular Diagnostics in BSI

The clinical value of rapid molecular diagnostics is determined by how results influence therapeutic decisions. Diagnostic speed alone does not consistently improve outcomes without timely modification of treatment [14,15]. Studies lacking structured clinical pathways report variable effects, highlighting the importance of coordinated implementation [74].

### 6.1. Measured Clinical Impact in BSI

Clinical benefit is best assessed using patient-centered outcomes rather than laboratory TAT. The most consistent effect among interventional studies is reduction in time to effective antimicrobial therapy. Rapid identification from positive BCs facilitates earlier treatment adjustment, particularly when supported by structured clinical response systems [14,62].

A network meta-analysis demonstrated lower mortality and shorter time to appropriate therapy when rapid diagnostics were integrated into care pathways compared with conventional approaches [75]. These findings suggest that outcome improvement depends on timely clinical action rather than diagnostic acceleration alone.

Implementation studies show similar patterns, including reduced exposure to broad-spectrum antibiotics and faster therapeutic optimization [74,76]. Effects on mortality and length of stay vary according to patient characteristics, infection severity, and resistance patterns.

The magnitude of benefit is greater in infections caused by resistant organisms, where early identification supports prompt initiation of active therapy. In susceptible infections, the relative effect is smaller, contributing to variation in reported outcomes [74,75].

### 6.2. Resistance-Guided Therapeutic Optimization

Rapid identification of resistance markers supports earlier refinement of empirical therapy. Molecular assays provide actionable information before completion of conventional susceptibility testing, allowing adjustment during the initial phase of management [15].

In Gram-positive infections, detection of methicillin resistance in *S. aureus* shortens the interval to targeted therapy. PCR-based approaches have demonstrated reduced time to appropriate treatment, with randomized trials confirming earlier therapeutic modification [14,77].

In Gram-negative infections, combined identification and resistance detection enable earlier selection of effective agents [76]. Delayed treatment in resistant infections is associated with worse outcomes, emphasizing the importance of timely adjustment [17].

These technologies also support de-escalation. Absence of resistance markers allows discontinuation of unnecessary broad-spectrum therapy, reducing antimicrobial exposure and selective pressure [78,79].

While rapid diagnostics support earlier therapeutic adjustment, their clinical impact varies depending on how results are integrated into decision-making [14,15]. In particular, resistance signals derived from molecular assays may not fully reflect expressed susceptibility, which can complicate early treatment choices in complex cases [72,73].

### 6.3. Translational Implications in CKD

In CKD, therapeutic decisions must account for altered drug handling and increased susceptibility to toxicity. Impaired renal function affects drug clearance and increases the risk of accumulation, especially with nephrotoxic agents [80,81].

Empirical regimens often include vancomycin or aminoglycosides, which are associated with kidney injury [47,82,83]. Earlier identification of causative pathogens enables transition to targeted therapy, limiting unnecessary exposure to these agents.

In *S. aureus* bacteremia, identification of methicillin susceptibility supports earlier use of β-lactam therapy instead of vancomycin, reducing nephrotoxic burden [77].

Among patients receiving HD, where antimicrobial exposure and infection burden are high, early detection of pathogens and resistance patterns supports both escalation in resistant infections and de-escalation when appropriate [7,15,74].

Empirical therapy commonly includes vancomycin combined with broad Gram-negative coverage, particularly in high-resistance settings [55,84]. Reduced unnecessary broad-spectrum exposure is associated with lower antimicrobial selective pressure [85,86].

Adjustment of antimicrobial dosing according to renal function is essential. Earlier microbiological confirmation facilitates appropriate selection and dosing, supporting safer and more individualized therapy [15,80]. Integration of microbiological results with pharmacologic dose adjustment is therefore essential, particularly in patients with limited physiologic reserve [87,88].

**Figure 3:**
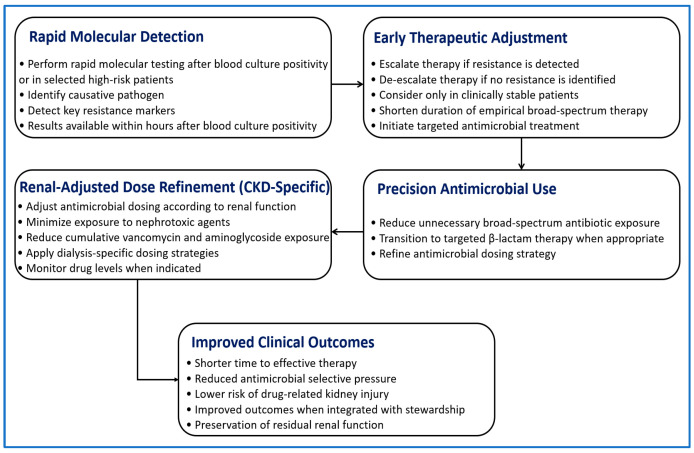
Clinical pathway showing how rapid molecular diagnostics support antimicrobial decision-making and renal safety in patients with CKD. Rapid testing allows early identification of pathogens and resistance markers, which guides timely adjustment of therapy. Detection of resistance supports escalation to active treatment, while absence of resistance allows cautious de-escalation in stable patients. Antimicrobial selection and dosing are adjusted according to renal function to reduce toxicity. This integrated approach helps optimize therapy, limit unnecessary broad-spectrum exposure, and improve clinical outcomes.

Although CKD-specific randomized evidence remains limited, studies in broader populations consistently show reduced time to effective therapy with rapid diagnostics [14]. The relationship between early identification, therapeutic adjustment, and treatment safety is illustrated in Figure 3.

### 6.4. Practical Clinical Integration in CKD and HD Settings

**Figure 4:**
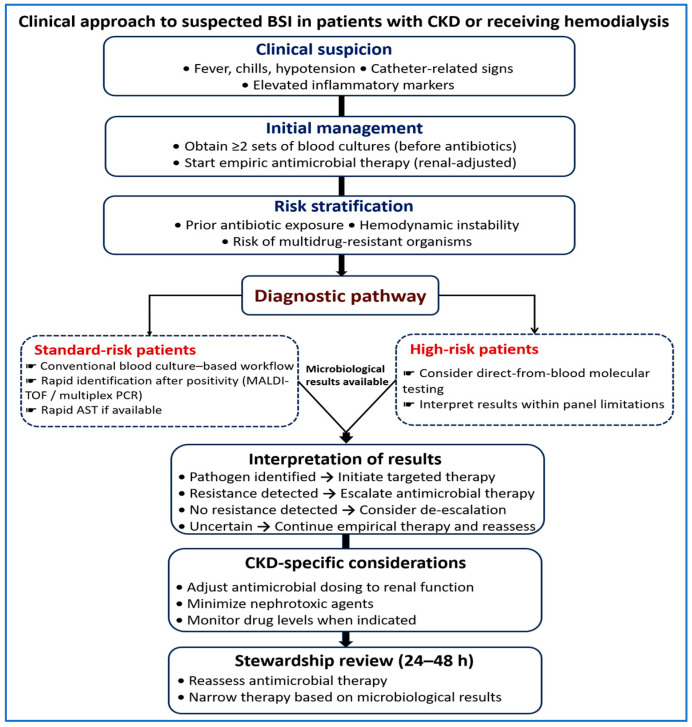
Clinical approach to suspected BSI in patients with CKD or receiving HD. This figure outlines a stepwise approach from clinical suspicion to antimicrobial optimization. BCs are obtained and empiric therapy is initiated early. Patients are stratified by clinical risk to guide the use of conventional diagnostics or adjunctive molecular testing. Microbiological results support targeted therapy, escalation when resistance is detected, or de-escalation when appropriate. Renal dosing and avoidance of nephrotoxic agents are considered throughout. Therapy is reassessed within 24–48 h based on microbiological results, resistance detection, and clinical response to guide escalation, de-escalation, or continuation of therapy.

Use of rapid molecular diagnostics should be guided by clinical context, infection severity, and likelihood of specific pathogens (Figure 4). BCs remain essential and should be obtained before antimicrobial therapy whenever possible [31,32].

Direct-from-blood assays may be considered in selected high-risk patients, particularly in cases of prior antimicrobial exposure or clinical instability. These methods provide early detection but are limited by predefined panels and should be applied selectively [33,34].

The most consistent benefit is observed after BC positivity. Rapid identification methods, including MALDI-TOF and multiplex PCR, shorten time to organism identification and support timely therapeutic adjustment when integrated into clinical care [14,62,75].

Diagnostic strategy should reflect clinical status. In stable patients with low pre-test probability, conventional culture with rapid testing after positivity is generally sufficient. In critically ill patients or those with prior antimicrobial exposure, adjunctive molecular testing may provide additional value.

Therapeutic decisions should be reassessed within 24–48 h based on combined clinical and microbiological data, with adjustment for renal function and toxicity risk.

In clinical practice, the choice of diagnostic platform should be guided by the patient’s condition and pre-test probability of specific pathogens. In stable patients with suspected BSI, conventional BC followed by rapid identification methods after culture positivity remains the preferred approach [14,62,75]. In critically ill patients or those with former antibiotic exposure, adjunctive molecular testing can allow earlier pathogen detection, although sensitivity varies [33,34]. Targeted PCR panels are most useful when common bacterial pathogens are suspected, whereas broader approaches such as metagenomic sequencing may be considered in selected cases with negative cultures or unclear etiology [67,68,69]. Rapid susceptibility testing methods can further support early therapeutic adjustment after organism identification [15].

Implementation in nephrology practice is influenced by logistical factors, particularly in outpatient dialysis settings. Access to advanced molecular platforms is often limited, and samples are frequently processed in centralized laboratories. As a result, TAT depends on transport and workflow in addition to assay performance.

Economic considerations also influence adoption. Molecular diagnostics typically involve higher upfront costs than conventional methods. However, earlier therapeutic optimization may reduce downstream costs related to prolonged hospitalization, inappropriate antimicrobial use, and complications. Cost-effectiveness depends on local infrastructure, patient population, and integration with clinical workflows.

## 7. Current Limitations, Evidence Gaps, and Implementation Challenges

Rapid molecular diagnostics reduce time to pathogen identification compared with conventional approaches. However, performance differs between platforms and clinical settings. Pooled analyses report moderate sensitivity with substantial heterogeneity, reflecting differences in assay design, patient populations, and study methodology [35]. These technologies therefore function as complementary tools rather than substitutes for culture-based diagnostics.

### 7.1. Analytical and Clinical Validation Gaps

Most prospective evaluations have been conducted in mixed hospital populations, including emergency department and inpatient cohorts [35,89,90]. Reported performance is influenced by assay characteristics, specimen handling, pathogen burden, and clinical context.

Current evidence primarily addresses pathogen detection relative to BC, with limited systematic evaluation of agreement between molecular resistance markers and phenotypic susceptibility testing [35,89,90]. As a result, available data support early organism identification but do not establish replacement of conventional susceptibility methods.

From a nephrology perspective, patients receiving maintenance HD experience a high burden of BSI, particularly in association with central venous catheters [91,92,93]. Despite this, prospective validation in CKD-specific populations remains scarce. Estimates derived from mixed cohorts may therefore not fully reflect performance in nephrology settings.

### 7.2. Interpretation Complexity

Sequencing-based approaches, including mNGS and plasma microbial cell-free DNA analysis, broaden the range of detectable pathogens beyond those recovered by culture [94,95,96]. Differences in sensitivity and specificity compared with culture are consistently observed.

Interpretation of sequencing results is influenced by variability in analytical thresholds, lack of standardization, and complexity of data processing [95]. Retrospective analyses report discordance with culture results, including findings suggestive of contamination or uncertain clinical significance [97]. These factors require integration of molecular results with clinical assessment and conventional microbiology.

Targeted multiplex platforms provide rapid detection of predefined organisms and selected resistance markers but remain limited by panel scope. Incomplete coverage and variable sensitivity support their use within combined diagnostic strategies rather than as stand-alone methods [35,89,90].

### 7.3. Implementation Considerations

Adoption of rapid molecular diagnostics requires financial resources, laboratory expertise, workflow adaptation, and coordination between clinical and laboratory teams. Real-world analyses identify infrastructure, cost, and integration into clinical pathways as key determinants of successful implementation [98]. Regulatory frameworks for advanced diagnostics continue to evolve and influence availability and uptake [99,100].

In dialysis settings, the high incidence of catheter-associated BSI underscores the need for reliable pathogen identification [91,93]. Early applications of sequencing-based methods in CKD populations demonstrate feasibility but also highlight challenges related to interpretation and operational complexity [101]. Further investigation is needed to define performance and feasibility in routine nephrology practice.

Overall, molecular diagnostics provide earlier microbiological information but show variability in accuracy and practical limitations. Their use is best considered within integrated diagnostic pathways alongside conventional BC. Key analytical, clinical, and implementation considerations are summarized in Table 3.

### 7.4. Comparative Performance and Diagnostic Inconsistencies Across Molecular Platforms

Direct comparison between molecular diagnostic platforms remains limited due to differences in study design and evaluation methods. Targeted PCR-based assays provide rapid detection of common pathogens but are inherently restricted to predefined panels, which may limit detection outside their scope [33,34,35,61]. In contrast, sequencing-based approaches allow broader, hypothesis-free detection, particularly in cases with negative cultures, but show greater variability in performance and require more complex interpretation [59,68,69].

A key limitation across platforms is inconsistency between genotypic resistance detection and phenotypic susceptibility. Although some resistance markers correlate well with expressed resistance, this relationship is not consistent across organisms or antimicrobial classes [72,73]. Discordance may occur when resistance mechanisms are not captured by diagnostic panels or when detected genes are not phenotypically expressed [70,72].

These differences highlight a practical limitation in clinical use. Molecular results alone may not provide a complete picture of antimicrobial susceptibility, particularly in complex infections. Their main value lies in early directional guidance rather than definitive resistance determination. Overall, current evidence indicates that diagnostic performance depends not only on the technology itself but also on the clinical context and the specific question being addressed.

## 8. Research Priorities and Future Directions

### 8.1. Diagnostic Validation in CKD

Patients receiving maintenance HD experience a high burden of BSI, with central venous catheters representing a major source of infection [91,92,93]. National surveillance data and cohort studies consistently report elevated infection rates and frequent catheter-related events requiring hospitalization and antimicrobial therapy [91,92,93]. These observations define a population in which accurate and timely pathogen identification is clinically relevant.

Despite this, rapid molecular diagnostic platforms have not been prospectively evaluated in cohorts composed exclusively of patients with CKD or maintenance dialysis. Existing evidence is derived largely from mixed hospital populations, with reported variability in sensitivity and specificity across studies [35]. Performance estimates from these settings may not reflect accuracy in nephrology practice. Stratified analyses by dialysis modality, vascular access type, and prior antimicrobial exposure would improve understanding of performance in this population.

Studies of metagenomic sequencing in CKD populations demonstrate feasibility and broader pathogen detection in selected settings [101]. However, these reports are descriptive and lack structured diagnostic accuracy assessment within defined dialysis cohorts. Key metrics, including sensitivity, specificity, predictive values, and time to actionable results, remain insufficiently characterized relative to BC as a reference standard.

Future investigations should include both analytical and clinical endpoints. Analytical evaluation should compare molecular methods directly with conventional BC, with results stratified by antimicrobial exposure and vascular access type. Clinical assessment should determine whether earlier identification translates into improved antimicrobial selection, timely de-escalation, and reduced exposure to nephrotoxic agents. Establishing this link is essential for defining the role of molecular diagnostics in dialysis practice.

### 8.2. Understanding Genotype-Phenotype Relationships in CKD

Evidence linking genotypic resistance detection to phenotypic susceptibility is derived from studies with variable methodology. Strong agreement has been demonstrated for selected resistance determinants in well-characterized organisms, primarily in controlled laboratory settings [102,103,104]. In broader analyses, predictive accuracy differs across species, resistance mechanisms, and analytical approaches [105,106].

Molecular diagnostics detect resistance through defined genetic markers, including *mecA* in *S. aureus*, extended-spectrum β-lactamase genes such as *CTX-M*, and carbapenemase genes in Gram-negative organisms. In general, clinical populations, detection of these markers often corresponds to phenotypic non-susceptibility [102,103,104]. Reporting agreement metrics and error rates relative to phenotypic standards is essential for safe clinical application.

Agreement is not consistent across all organisms or antimicrobial classes. Resistance phenotypes may result from regulatory variation, chromosomal mutations not captured by standard panels, altered gene expression, or permeability changes. Differences in bioinformatic pipelines and database curation further influence prediction accuracy [106]. These factors require validation against phenotypic reference methods before clinical reliance.

Data specific to dialysis populations are limited. Available reports describe detection of resistance genes such as *CTX-M* and *vanA* in isolates that also exhibit phenotypic resistance, but these observations are derived from small cohorts [107]. Many CKD-focused studies report phenotypic resistance patterns without corresponding molecular analysis or formal concordance assessment [108,109].

This limitation has practical consequences. Patients with impaired renal function are vulnerable to both delayed effective therapy and drug-related toxicity. Inaccurate resistance prediction may either delay active treatment or prolong exposure to nephrotoxic agents. Genomic resistance studies emphasize the need to report sensitivity, specificity, and error rates relative to phenotypic standards before clinical implementation [103,106].

This gap has practical implications. Inaccurate resistance prediction may delay effective therapy or prolong exposure to nephrotoxic agents in patients with impaired renal function. Future research should include structured concordance studies comparing genotypic and phenotypic resistance across CKD and dialysis cohorts, with evaluation by organism, resistance mechanism, antimicrobial exposure, and vascular access type. Until more robust data are available, genotypic resistance findings should complement, rather than replace, phenotypic testing and clinical assessment.

### 8.3. Integration with Antimicrobial Stewardship in Dialysis Units

Patients undergoing chronic HD experience frequent infections and repeated antimicrobial exposure. Observational studies report common prescribing errors related to indication, dose, and duration in outpatient dialysis settings [110]. Surveillance data also show elevated BSI rates, particularly among patients with central venous catheters [91]. These patterns support the need for structured antimicrobial stewardship in this population.

Antimicrobial stewardship programs focus on optimizing selection, dosing, and duration of therapy. Core components include multidisciplinary oversight, facility-specific guidance, prospective audit with feedback, and monitoring of antibiotic use [111]. These principles can be adapted to dialysis workflows and patient-specific risks.

In broader BSI populations, combining rapid diagnostics with structured stewardship consistently improves antimicrobial use and reduces time to effective therapy. Studies report earlier therapeutic adjustment, reduced hospital stay, and, in some settings, lower mortality compared with conventional management [112,113]. These findings reveal that diagnostic advances achieve greater clinical impact when integrated into coordinated stewardship processes.

Evidence specific to dialysis units remains limited. Few studies evaluate how rapid diagnostics influence prescribing behavior, resistance patterns, or outcomes in maintenance HD populations. Dialysis-specific factors, including altered pharmacokinetics, vascular access-related infection risk, and repeated antimicrobial exposure, support the need for tailored stewardship strategies.

Current literature in dialysis settings primarily describes prescribing patterns and infection surveillance rather than interventional outcomes. In contrast, stronger evidence is available from general BSI cohorts. Relevant stewardship approaches are summarized in Table 4.

## 9. Conclusions

Rapid molecular diagnostics expand the ability to detect pathogens and resistance determinants in BSI. In patients with CKD, their application is particularly relevant given the complexity of antimicrobial selection and the need to minimize drug-related toxicity. Current platforms differ in accuracy and scope, and genetic resistance markers do not consistently correspond to phenotypic susceptibility. These constraints require careful interpretation alongside microbiological results and clinical context. Available evidence is largely derived from mixed patient populations, and data specific to nephrology settings remain insufficient. Defining diagnostic performance, resistance concordance, and clinical benefit in CKD-focused cohorts remains a priority. In clinical practice, these technologies can support more precise antimicrobial use when applied within structured care pathways that account for renal function and treatment safety.

## Figures and Tables

**Table 1:** Limitations of conventional diagnostic methods in BSI among kidney patients.

Method	Time to Result	Resistance Detection Capability	Clinical Limitation in CKD	Clinical Decision Implication	References
Automated BC	12–48 h to initial positivity; ≥72 h in low-burden infections	Requires organism growth; additional incubation for susceptibility testing	Delayed targeted therapy; increased risk in clinically unstable patients	Continue empirical therapy with clinical monitoring	[1,3,36]
BC after prior antibiotic exposure	Reduced culture yield when antibiotics given before sampling	Resistance testing limited if organisms are not recovered	Infection may be missed; empirical therapy may be prolonged	Negative cultures do not exclude infection; interpret clinically	[31,32]
BC for *Candida* species	Longer time to positivity; reported sensitivity approximately 50–75%	Resistance profiling possible only after organism growth	Low fungal burden may delay detection and treatment	Consider empirical antifungal therapy in high-risk patients	[38,39]
Phenotypic AST	Additional 24–48 h after culture positivity	Detects expressed resistance after incubation	Delayed results prolong broad-spectrum therapy and nephrotoxic exposure	Adjust therapy based on results; balance efficacy and toxicity	[42,46,47]
BC in catheter-related infection	Detection influenced by intermittent bacteremia	Resistance determined only after organism recovery	Biofilm-related infection may delay confirmation	Repeat cultures and evaluate catheter-related source	[27,49,50,51]
BC with CNS	Requires evaluation of multiple sets	Resistance data may reflect contamination rather than true infection	Diagnostic uncertainty may delay treatment decisions or lead to unnecessary therapy	Interpret results in clinical context to distinguish contamination from true infection	[1,36,50]

**Table 3:** Documented limitations and practical considerations of rapid molecular diagnostics for BSI.

Key Issue	What the Published Evidence Shows	Clinical Implication	References
Variation in diagnostic performance	Sensitivity varies between platforms with substantial between-study heterogeneity	Negative results do not exclude infection; interpret in clinical context	[35,90,94]
Continued role of BC	Molecular assays miss some pathogens; BC remains essential	BC required for confirmation and full susceptibility testing	[35]
Resistance determination	Focus on detection; limited data on concordance with phenotypic susceptibility	Resistance results require cautious interpretation and phenotypic confirmation	[35,90,94]
Study populations	Evidence mainly from mixed hospital cohorts rather than CKD-specific populations	Applicability to CKD remains uncertain; interpret with caution	[35,90,94]
Interpretation of sequencing results	Interpretation depends on thresholds, standardization, and clinical context	Interpret results alongside clinical findings	[95,96]
Discordance and contamination	Discordance with culture occurs; contamination reported in some cases	Interpret unexpected results with caution	[97]
Laboratory adoption	Implementation depends on resources, expertise, and workflow integration	Effective use requires institutional support and integration	[98]
Regulatory environment	Regulatory frameworks influence adoption and clinical use	Availability and use vary through healthcare settings	[99,100]

**Table 4:** Antimicrobial stewardship interventions relevant to dialysis units and BSI Management.

Intervention	Population	Study Design	Main Findings	Clinical Relevance	References
Evaluation of antibiotic prescribing practices with stewardship recommendations	Maintenance HD patients	Observational study	Frequent inappropriate prescribing; stewardship improves antimicrobial use	Supports structured antimicrobial stewardship in dialysis settings	[110,111]
National surveillance of BSIs in outpatient dialysis facilities	Outpatient HD facilities	National surveillance analysis	High BSI rates, especially with catheter use; need for surveillance and prevention	Emphasizes surveillance and infection prevention in dialysis units	[91]
Rapid pathogen identification combined with active antimicrobial stewardship intervention	Hospitalized patients with BSI	Quasi-experimental study	Reduced time to therapy, length of stay, mortality, and healthcare costs	Supports integrating rapid diagnostics with stewardship to improve outcomes	[112]
Rapid diagnostic testing integrated with pharmacist-led stewardship review	Hospitalized patients with BSI	Retrospective cohort study	Earlier identification, increased de-escalation, shorter stay, and lower mortality	Supports multidisciplinary stewardship to optimize antimicrobial use	[113]
Rapid diagnostic testing within stewardship-integrated workflows	Mixed inpatient populations	Systematic review	Earlier identification and improved antimicrobial optimization with stewardship integration	Highlights benefit of diagnostics integrated with stewardship programs	[35]

## Data Availability

No new data were created or analyzed in this study. Data sharing is not applicable.
